# Reviewer Acknowledgement

**DOI:** 10.1186/s13064-015-0030-1

**Published:** 2015-03-15

**Authors:** Chris Q Doe, William Harris, Kang Shen, Rachel Wong

**Affiliations:** University of Oregon, United States of America, ᅟ, ᅟ; University of Cambridge, United Kingdom, ᅟ, ᅟ; Stanford University, United States of America, ᅟ, ᅟ; University of Washington, United States of America, ᅟ, ᅟ

## Abstract

The editors of Neural Development would like to thank all the reviewers who have contributed to the journal in Volume 9 (2014).

**Clare Baker**

United Kingdom

**Laure Bally-Cuif**

France

**Linda Barlow**

United States of America

**Martin Bastmeyer**

Germany

**Renata Basto**

France

**David Berson**

United States of America

**Florence Besse**

France

**Paola Bovolenta**

Spain

**Robert Bradley**

United States of America

**Jean-François Brunet**

France

**Noel Buckley**

United Kingdom

**Florencia Cavodeassi**

Spain

**Frederic Charron**

Canada

**Alain Chedotal**

France

**Matthew Colonnese**

United States of America

**Helen Cooper**

Australia

**Michael Crair**

United States of America

**Igor B Dawid**

United States of America

**Luca Della Santina**

Italy

**Claude Desplan**

United States of America

**Richard Dorsky**

United States of America

**Wolfgang Driever**

Germany

**Judith S Eisen**

United States of America

**Heithem El-Hodiri**

United States of America

**Kazuo Emoto**

Japan

**Johan Ericson**

Sweden

**James Fawcett**

United Kingdom

**Donna Fekete**

United States of America

**Marla Feller**

United States of America

**Monica Folgueira**

United Kingdom

**Troy Ghashghaei**

United States of America

**Tom Glaser**

United States of America

**Cayetano Gonzalez**

Spain

**Ray Guillery**

United Kingdom

**Volker Hartenstein**

United States of America

**Samer Hattar**

United States of America

**Eloisa Herrera**

Spain

**Simon Hippenmeyer**

Austria

**Christine Holt**

United Kingdom

**Andrew Huberman**

United States of America

**Suresh Jesuthasan**

Singapore

**Ryoichiro Kageyama**

Japan

**Hiroyuki Kamiguchi**

Japan

**Raymond Keller**

United States of America

**Tim Kennedy**

Canada

**Eunjoon Kim**

Korea, South

**Christian Klaembt**

Germany

**Ruediger Klein**

Germany

**Alex Kolodkin**

United States of America

**Lawrence Kromer**

United States of America

**Ronald Kühnlein**

Germany

**Guofa Liu**

United States of America

**Hernan Lopez-Schier**

Germany

**Liqun Luo**

United States of America

**Giuseppe Lupo**

Italy

**David Lyons**

United Kingdom

**David Miller**

United States of America

**William Moody**

United States of America

**Donald Francis Newgreen**

Australia

**Laurent Nguyen**

Belgium

**Shigeo Okabe**

Japan

**Hitoshi Okamoto**

Japan

**Suzanne Paradis**

United States of America

**Juha Partanen**

Finland

**Gang Peng**

China

**Muriel Perron**

France

**Andreas Prokop**

United Kingdom

**Pamela Raymond**

United States of America

**Sylvie Retaux**

France

**Anne Ridley**

United Kingdom

**Carol Schuurmans**

Canada

**Nenad Sestan**

United States of America

**Jane C Sowden**

United Kingdom

**Esther Stoeckli**

Switzerland

**Guy Tear**

United Kingdom

**Kristin Tessmar-Raible**

Austria

**Andrew Tomlinson**

United States of America

**Eric Turner**

United States of America

**David Van Vactor**

United States of America

**Fan Wang**

United States of America

**Stephen Wilson**

United Kingdom

**Xiang Yu**

China

**Kai Zinn**

United States of America

**Karen Zito**

United States of America

**Michael Zuber**

United States of America

